# *Platynosomum illiciens* infection in domestic cats: insights from a sanctuary

**DOI:** 10.29374/2527-2179.bjvm005023

**Published:** 2023-12-04

**Authors:** Bruno Alberigi, Mateus Daudt Matos, Thaís Ribeiro Correia, Bruno de Oliveira Telles Ferreira, Lais Sperandio Cassani, Roxanne Marina da Silva Roque, Sidney Jiro Nohara, Norma Labarthe

**Affiliations:** 1 Veterinarian, DSc. Departamento de Medicina e Cirurgia Veterinária (DMCV), Instituto de Veterinária (IV), Universidade Federal Rural do Rio de Janeiro (UFRRJ). Seropédica, Seropédica, RJ, Brazil.; 2 Veterinarian PPGMV, IV, UFRRJ. Seropédica, Seropédica, RJ. Brazil.; 3 Veterinarian, DSc. Departamento de Parasitologia Animal (DPA), IV, UFRRJ. Seropédica, Seropédica, RJ, Brazil.; 4 Veterinarian, Resident. Programa de Residência em Medicina Veterinária - Diagnóstico em Parasitologia Animal. IV, UFRRJ. Seropédica, Seropédica, RJ, Brazil.; 5 Veterinarian, MSc. Autonomus, Rio de Janeiro, RJ, Brazil; 6 Veterinarian, DSc., Programa de Pós-graduação Bioética, Ética e Saúde Coletiva, Fundação Oswaldo Cruz (Fiocruz), Rio de Janeiro, RJ, Brazil

**Keywords:** cats, liver fluke, bile, gatos, verme do fígado, bile

## Abstract

*Platynosomum illiciens*, a trematode parasite known for its intricate life cycle, predominantly infests the liver, gallbladder, and bile ducts of domestic cats. In this study, we examined feline fecal samples from a cat sanctuary in Metropolitan Rio de Janeiro, Brazil, aiming to determine whether, even in such environments, cats retain their atavistic hunting habits, as evidenced by the presence of *P. illiciens* in fecal samples. The infection diagnosis utilized centrifugal sedimentation in the formalin-ethyl acetate test. Out of 72 fecal samples collected from various cats, four tested positive for *P. illiciens* eggs. This study serves as a reminder that even in environments where they are well-fed, cats exhibit predatory behavior, engaging in hunting and consuming prey, thus exposing themselves to parasites. It emphasizes the importance of veterinarians and cat caretakers being cognizant of the widespread presence of *P. illiciens* in Brazil and considering it in the differential diagnosis for cats presenting with liver or gallbladder issues. In conclusion, our findings underscore that the ancestral instinct for hunting and predation, preserved in domestic cats, persists despite fulfilling their nutritional needs.

## Resumo

*Platynosomum illiciens*, um parasito trematódeo conhecido por seu ciclo de vida complexo, infesta predominantemente o fígado, a vesícula biliar e os ductos biliares de gatos domésticos. Neste estudo, examinamos amostras fecais de felinos de um santuário de gatos na região metropolitana do Rio de Janeiro, Brasil, com o objetivo de determinar se, mesmo nesses ambientes, os gatos mantêm seus hábitos de caça, evidenciado pela presença de *P. illiciens* em amostras fecais. O diagnóstico da infecção foi realizado por sedimentação centrífuga no teste de formalina-acetato de etila. Foram obtidas 72 amostras fecais de diferentes gatos e quatro testaram positivo para ovos de *P. illiciens*. Este estudo é um lembrete de que os gatos terão comportamento predatório, caçando e consumindo presas mesmo quando bem alimentados, ficando expostos ao parasito. Veterinários e cuidadores de gatos devem estar atentos à ampla presença de *P. illiciens* no Brasil e considerá-la no diagnóstico diferencial de gatos com problemas de fígado ou vesícula biliar. Concluindo, nossos resultados mostram que o instinto atávico dos gatos que se mantiveram conservados durante a domesticação não pode ser anulado quando as necessidades nutricionais dos gatos são satisfeitas.

## Introduction

*Platynosomum illiciens* predominantly targets the liver, gallbladder, and bile ducts of its final hosts, leading to the shedding of eggs into the environment. Cats become infected when they hunt and ingest an infected paratenic hosts, such as snails or lizards (Basu & Charles, 2014). Despite efforts by pet cat owners to deter hunting behavior by providing a diverse and high-quality diet, including dry and moist cat food with various flavors and textures, as well as employing food puzzles to meet their cats’ sophisticated demands and offer environmental enrichment (Delgado et al., 2020; Dodd et al., 2020), the instinctual drive to hunt may persist. This study focuses on cats in a sanctuary where feline residents receive care akin to that provided to pets. The investigation aims to determine whether, even in such environments, cats retain their atavistic hunting habits, as evidenced by the presence of *P. illiciens* in fecal samples.

## Material and methods

The cat sanctuary surveyed spans a 13,142 m^2^ area in Metropolitan Rio de Janeiro, RJ, accommodating approximately 700 neutered and microchipped cats. These feline residents enjoy unrestricted access to both open spaces and indoors rooms. Clean running water bowls are consistently available, and premium dry food is provided *ad libitum* in the morning. In the late afternoon, moist canned food becomes accessible to all cats, followed by another round of *ad libitum* dry food.

Upon obtaining formal consent, fecal samples from various animals were collected after natural defecation. The analysis employed an adapted centrifugal sedimentation technique utilizing the adapted formalin-ethyl acetate test (Erdman, 1981; Monteiro, 2017; Palumbo et al. 1976; Ritchie, 1948).

## Results

**Figure 1:**
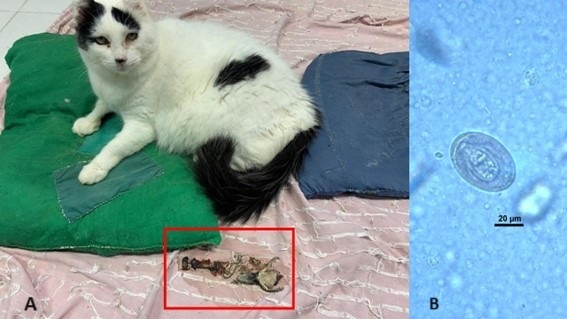
(A) A well-nourished cat tending to its partially eaten game, a lizard (highlighted in the red rectangle). (B) *Platynosomum illiciens* egg from the cat (40× magnification).

A comprehensive examination of 72 fecal samples from different cats revealed that four samples tested positive for *P. illiciens* eggs (5.5%) (Figure 1).

## Discussion

The parasite *P. illiciens* is widely recognized in Brazil for causing subclinical infections. Clinical manifestations, when present, typically manifest as hepatic and biliary pathology (Azevedo et al., 2012; Camatta Campos et al., 2018; Ferraz et al., 2021, 2022; Lima et al., 2021; Salomão et al., 2005). While previous reports highlight the presence of this infection in the domestic cat population of Rio de Janeiro (Azevedo et al., 2012; Salomão et al., 2005), comprehensive surveys on its frequency for meaningful comparisons are lacking.

This study, focusing on well-cared for cats in a sanctuary, reveals that even in such environments, where nutritional needs are met, cats engage in hunting and consuming prey. This behavior exposes them to *P. illiciens* infection, indicating that they can supplement their diet by hunting, potentially leading to infection with this parasite, and yet appear asymptomatic. Veterinarians and cat caretakers should be vigilant, recognizing the widespread presence of *P. illiciens* in Brazil and acknowledge that cats, despite provisions, may engage in hunting (Delgado et al., 2020). Clinicians handling cases of liver or gallbladder issues in cats must consider *P. illiciens* as a differential diagnosis. The intermittent shedding of the parasite’s eggs with feces necessitates specialized coproparasitological tests for accurate detection (Basu & Charles, 2014; Lima et al., 2021; Silva et al., 2023).

## Conclusions

Our findings underscore that the atavistic instinct preserved in domestic cats persists, unaffected by the fulfillment of their nutritional needs.
